# Comprehensive *in vitro* and *in vivo* evaluation of therapeutic potential of Bacopa-derived asiatic acid against a human oral pathogen *Streptococcus mutans*

**DOI:** 10.3389/fmicb.2024.1404012

**Published:** 2024-06-25

**Authors:** Rajendran Jeyasri, Pandiyan Muthuramalingam, Arumugam Priya, Rajaiah Alexpandi, N. R. Siva Shanmugam, Saminathan Nivetha, Hyunsuk Shin, Shunmugiah Karutha Pandian, Arumugam Veera Ravi, Manikandan Ramesh

**Affiliations:** ^1^Department of Biotechnology, Science Campus, Alagappa University, Karaikudi, Tamil Nadu, India; ^2^Division of Horticultural Science, College of Agriculture and Life Sciences, Gyeongsang National University, Jinju, South Korea; ^3^Department of Medicine, Division of Gastroenterology and Hepatology, Pennsylvania State University College of Medicine, Hershey, PA, United States; ^4^School of Materials Science and Engineering, Zhejiang Sci-Tech University, Hangzhou, China; ^5^Department of Food Science and Technology, Nebraska Food for Health Center, University of Nebraska - Lincoln, Lincoln, NE, United States

**Keywords:** acidogenicity, asiatic acid, *Bacopa monnieri*, *Danio rerio*, dental caries, *Streptococcus mutans*

## Abstract

Dental caries is a common human oral disease worldwide, caused by an acid-producing bacteria *Streptococcus mutans*. The use of synthetic drugs and antibiotics to prevent dental caries has been increasing, but this can lead to severe side effects. To solve this issue, developing and developed countries have resorted to herbal medicines as an alternative to synthetic drugs for the treatment and prevention of dental caries. Therefore, there is an urgent need for plant-derived products to treat such diseases. *Bacopa monnieri*, a well-documented medicinal plant, contains 52 phytocompounds, including the pentacyclic triterpenoid metabolite known as asiatic acid (ASTA). Hence, this study aimed to demonstrate, for the first time, the antibacterial activity of phytocompound ASTA against *S. mutans*. The findings revealed that ASTA significantly inhibited the growth of *S. mutans* and the production of virulence factors such as acidurity, acidogenicity, and eDNA synthesis. Molecular docking analysis evaluated the potential activity of ASTA against *S. mutans* virulence genes, including *VicR* and *GtfC*. Furthermore, toxicity assessment of ASTA in human buccal epithelial cells was performed, and no morphological changes were observed. An *in vivo* analysis using *Danio rerio* (zebrafish) confirmed that the ASTA treatment significantly increased the survival rates of infected fish by hindering the intestinal colonization of *S. mutans*. Furthermore, the disease protection potential of ASTA against the pathognomonic symptom of *S. mutans* infection was proven by the histopathological examination of the gills, gut, and kidney. Overall, these findings suggest that ASTA may be a promising therapeutic and alternative drug for the treatment and prevention of oral infection imposed by *S. mutans*.

## 1 Introduction

In ancient times, plants used for their curative potential against any form of disease were termed as medicinal plants. *Bacopa monnieri*, an ancient Indian medicinal herb, is majorly used for the treatment of various neurological diseases/disorders due to its several pharmacologically active potential phytocompounds (Sekhar et al., 2019). *B. monnieri* belongs to the family of Plantaginaceae, is widely extended throughout the Indian subcontinent, and is used as a brain tonic for memory enhancement, concentration, skin disorders, and various other ailments (Kumar et al., 2016). Overall, to date, 52 biologically active phytocompounds were reported in *B. monnieri* (Shefin et al., 2016; Jeyasri et al., 2020); among them, bacoside A and B are the two main phytocompounds that have memory-enhancing properties. The plant *Bacopa* exhibits various pharmacological properties including anti-fungal (Fazlul et al., 2019), anti-epileptic (Komali et al., 2021), antidepressant (Mannan et al., 2015; Brimson et al., 2021), anti-bacterial (Fazlul et al., 2019), antioxidant (George, 2017), anticancer (Ghosh et al., 2021), analgesic (Hossain et al., 2012), hepatoprotective (Karim et al., 2020), and anti-anticonvulsant (Kaushik et al., 2009) ones.

*Streptococcus mutans* is a major causative agent for human dental caries. Various known factors that are associated with plaque formation include microbial adherence to the tooth surface, enzyme activity, and acid precipitation (Leach and Hayes, 1968; Gibbons and van Houte, 1980). *S. mutans* has the potential to form biofilms known as dental plaque on tooth surfaces (Kawabata and Hamada, 1999). *S. mutans* synthesizes glucans with the enzyme reaction catalyzed by glucosyltransferases (GTFs) from the dietary sucrose, facilitating adhesion and colonization of bacteria to the tooth surfaces (Yamashita et al., 1993). Furthermore, *S. mutans* contain a significant cell surface protein called protein antigen c (PAc), which contributes to its virulence by promoting bacterial adhesion through its interaction with salivary pellicles and the formation of dental caries (Koga et al., 1990). Altogether, these surface proteins together generate tooth plaque, thus causing dental caries.

In the current era, dental caries is the most common human oral disease globally. The World Health Organization (WHO) revealed that approximately 60–90% of children and 64% of older adults have dental caries. Hence, there is an alarming need to improve the therapeutic and diagnostic treatment of dental caries, especially childhood caries. Moreover, the use of synthetic drugs and antibiotics has increased gradually, particularly in children, and it causes severe ill effects such as tooth discoloration, vomiting, diarrhea, stomach burning, skin itching, gastrointestinal disorders, and sore tongue and mouth (Macy, 2015; Olveira and González-Molero, 2016). To overcome these problems, most of the people started using herbal medicines as alternatives to synthetic drugs. Evidence revealed that, in developing countries, approximately 80% of people prefer medicinal plants for their dental treatment (Calixto, 2000). In United States, ~12% of people use alternative medicine and a meager 5% prefer plant-derived treatments (Barnes et al., 2008).

Several studies have explored the therapeutic benefits of many active phytochemical plants in the treatment of tooth decay (Cruz Martinez et al., 2017). Singh and Dhakre (1989) reported the use of *Ocimum basilicum* L. (Lamiaceae), *Argemone maxicana* L. (Papaveraceae), and *Azadirachta indica* A. Juss. (Meliaceae) for plaque reduction and modified gingival scores. Kanwar et al. (2006) reported the beneficial effects of *Achyranthes aspera* L., *Aegle marmelos* L. Correa, and *Vitex negundo* L. to treat and manage dental decay. Gum bleeding has been found to be prevented by the use of *Juglens regia* L., *Zanthoxylum armatum*, and *Caram carvi* L. (Tomar, 2007; Sharma and Joshi, 2008). Medicinal plants and their bioactive compounds have several significant properties that have proven to be beneficial for oral hygiene. There are several topically applicable phytochemicals used for the management and treatment of oral diseases.

Among the 52 compounds from *B. monnieri*, asiatic acid (ASTA) is one of the potential phytocompounds based on the pharmaceutical bioactive scores. ASTA is a crucial bioactive compound, and it has been studied for its various therapeutic activities such as antioxidant, anti-inflammatory and neuroprotective effects. Asiatic acid has been predominantly investigated within a therapeutic framework; however, its pharmacological properties also provide a basis for potential preventive applications, especially in chronic conditions impacted by inflammation and oxidative stress. Their specific application (whether preventative or therapeutic) within clinical environments would depend on further research findings and the specific health conditions under consideration. The anti-inflammatory, gastroprotective, antiulcer, cardiovascular, hapatoprotective, hypolipidemic, anti-HIV, anti-atherosclerotic, and immunoregulatory properties of ASTA have already been reported (Cho et al., 2006; Jeong et al., 2007; Wojnicz et al., 2013). Only very few reports are available for the pharmaceutical and anti-bacterial properties of ASTA. However, no data on the anti-infective potential of ASTA against *S. mutans* infection *in vivo* have been reported to date. With this background, this pilot study for the first time intended to evaluate the antibacterial and anti-infective potential of ASTA against the *S. mutans* infection. In addition, this study demonstrates its expanded therapeutic potential against *S. mutans*.

## 2 Materials and methods

### 2.1 Ethical statement

For the present investigation, human buccal epithelial cells (HBECs) were obtained from individuals with good oral health for the assessment of toxicity. For the collection of HBECs, individuals with good oral health were requested to carefully scrape the buccal surface inside their cheeks. The experimental designs and use of HBECs were evaluated and endorsed by the Institutional Ethical Committee, Alagappa University, Karaikudi (IEC Ref No: IEC/AU/2018/5). All methods were carried out in accordance with the relevant guidelines and regulations.

### 2.2 Bacterial strain and growth conditions

The *Streptococcus mutans* UA159 strain was acquired from the American Type Culture Collection (ATCC 700610) for our study. *S. mutans* was cultured in Todd Hewitt Broth (THB) (Hi-media, Mumbai, India) with 1% of sucrose and yeast extract (THYES) under anaerobic conditions at 37°C overnight.

### 2.3 Phytocompounds

ASTA, the phytocompound used for the evaluation of antibacterial activity, was purchased from Thermo Fisher Scientific, USA, and the stock solution was prepared in dimethyl sulfoxide (DMSO) solution (50 mg/mL) and stored at 4°C.

### 2.4 Determination of minimum inhibitory concentration

The MIC of ASTA against planktonic *S. mutans* was assessed using the broth dilution method as reported by the Clinical and Laboratory Standards Institute (CLSI) (Wikler, 2006). The cell density (2 × 10^6^ cells/mL) of *S. mutans* was used in the present study. Precisely, 10 μL of *S. mutans* suspension was added in 1 mL of sterile THYES broth in a microtiter plate (MTP) supplemented with ASTA at concentrations ranging from 2 to 1024 μg/mL. Appropriate vehicle and negative controls were maintained parallelly. Followed by incubation, the cell density was calculated at OD_600nm_ by using a multifunctional spectrophotometer (Spectra Max 3, Molecular Devices, USA).

### 2.5 Determination of minimum bactericidal concentration

Determination of the MBC and growth inhibitory effect of ASTA against *S. mutans* was confirmed by spot assay and CFU. In brief, 10 μL of *S. mutans* culture was incubated with various concentrations of ASTA that were determined using the MIC assay. At the end of incubation, both treated and untreated cells were used for serial dilution, and the dilutions were spread, plated, and spotted on agar plates (Todd-Hewitt Agar -THA) and incubated at room temperature for 24 h. Finally, the results were archived by the gel documentation system (Bio-Rad Laboratories, XR+, USA).

### 2.6 Effects of ASTA on *S. mutans* biofilms

#### 2.6.1 Quantification and microscopic visualization

To evaluate the impact of ASTA on biofilm formation, spectrophotometric and microscopic analyses were performed. For this biofilm assay, different concentrations of ASTA in 24 MTP were used and incubated at room temperature overnight. After incubation, the supernatant was removed, and the biofilms cells attached on the surface in MTP were washed twice with sterilized phosphate-buffered saline (PBS) solution to eliminate loosely adherent cells. The surface-bound biofilm cells were stained and destained by 0.4% crystal violet (CV) and 15% glacial acetic acid, respectively. Furthermore, the destained solution was measured at OD_570nm_ using a multifunctional UV–Vis spectrophotometer (Spectra Max 3, Molecular Devices, USA).

For microscopic observation, the biofilm assay was performed on glass slides (1 x 1 cm) with different concentrations of ASTA (8, 16, and 32 μg/mL) and incubated at room temperature for 24 h. The following day, the glass slides were carefully taken out and gently rinsed with distilled water. The slides were stained using 0.4% CV and air-dried for 10 min. Finally, the stained biofilms cells in the slides were observed under a light microscope (Nikon Eclipse 80i, USA) at 400 X magnification.

#### 2.6.2 Mature biofilm disruption

To examine the impact of biofilm disruptive efficiency of ASTA, *S. mutans* was permitted to develop mature biofilms. After overnight incubation, planktonic biofilm cells were eliminated and fresh THYES broth was added with different concentrations of ASTA and incubated for 24 h. After the incubation, the cells were scraped out from the surface and mixed thoroughly with the PBS solution. Furthermore, the cells were serially diluted, distributed evenly on agar plates using the spread plated method, and incubated at 37°C for 24 h. Furthermore, the numbers of viable cells were observed in control and treated plates. On the other hand, microscopic visualization of mature biofilm disruption was carried out with the help of glass slides (1 x 1 cm). The glass slides were gently washed with distilled water and air-dried. After air-drying, glass slides were stained with 0.4% CV and visualized under a light microscope at 400 X magnification (Nikon Eclipse 80i, USA), and the images were generated with the help of an attached digital camera.

#### 2.6.3 Acidogenicity potential of ASTA against *S. mutans*

Glycolytic pH drop assay was carried out to analyze the impact of ASTA on the acidogenic properties of *S. mutans*. For this assay, the harvested *S. mutans* cells were washed with PBS and resuspended with a salt solution composed of potassium chloride and magnesium chloride (50 mM KCl + 1 mM MgCl_2_) with different concentrations of ASTA (2 to 128 μg/mL). To this salt solution, 1% glucose (w/v) was added, and the pH of the mixture was adjusted between 7.2 and 7.4 by adding potassium hydroxide (0.2 M). The pH reduction through the glycolytic potential of *S. mutans* was observed for every 15-min intervals for a period of 120 min (Priya et al., 2021).

#### 2.6.4 pH of the spent medium

For the measurement of acidity, 10 μL of *S. mutans* culture was added to the THYES broth (pH 7.2) supplemented with the absence and presence of ASTA at different concentrations. The pH of the medium was determined following the inclusion of ASTA and subsequently 24 h of incubation.

#### 2.6.5 Effect of ASTA on the acid tolerance of *S. mutans*

The impact of ASTA on the acid tolerance potential of *S. mutans* was evaluated by counting the viable cells after exposure to pH levels of mild and extremely acidic conditions. *S. mutans* cells were harvested with various concentrations of ASTA and collected by centrifugation. After centrifugation, the pellet was divided into two aliquots: one for adapted cells and another one for unadapted cells. The unadapted *S. mutans* cells were resuspended in THYES broth at an adverse pH value of 3.5 and incubated at room temperature for 2 h, whereas the adapted cells were primarily cultured in broth at mild pH 5.5, incubated for 1 h at 37°C. At the end of the incubation period, the cells were immediately transferred to the broth at a lethal pH 3.5 for 2 h at 37°C. Followed by incubation, the number of viable cells obtained from the unadapted and adapted cells was observed through CFU analysis by serial dilution and plating on agar plates at 37°C for 24 h (Priya et al., 2021).

#### 2.6.6 Influence of ASTA on eDNA content in the *S. mutans* biofilm

The presence of eDNA in *S. mutans* biofilms was examined through agarose gel electrophoresis (AGE). In a sterile polystyrene six-well plate, the *S. mutans* was allowed to form biofilms in various concentrations of ASTA (2, 4, 8, and 16 μg/mL) and incubated at 37°C for 24 h. Following the incubation period, the planktonic and loosely adherent cells were removed carefully without disturbing the surface-attached biofilms and rinsed twice with PBS solution. Then, the surface-attached biofilms cells were scraped out and resuspended in Tris-EDTA (TE) buffer (containing 10 mM Tris and 1 mM EDTA, pH 8.0). Then, the collected biofilms were subjected to vigorous vortexing for 30–40 min to detach the complex mixture of the cells. After vortexing, the biofilm cells were centrifuged, and the supernatant was collected. Finally, the presence of eDNA content in the supernatant was observed through AGE (1.5% w/v), followed by ethidium bromide staining (Kaplan et al., 2012), and visualized through the gel documentation system (Bio-Rad Laboratories, XR+, USA).

### 2.7 Toxicity assessment on human buccal epithelial cells

To check the toxicity of ASTA on HBECs, orally healthy individuals were selected. For the collection of HBECs, healthy individuals were instructed to use a sterile cotton swab to gently scrape the buccal surface of the cheeks. The swabs were sequentially suspended in sterilized PBS solution, which was used promptly. The collected individual buccal cells were pooled and then centrifuged at 5,000 rpm for 7 min. The pellet was washed twice with PBS solution to eliminate debris. After removing debris, the cells were incubated with different concentrations of ASTA at 37°C for 30 min to evaluate the detrimental effects. Hydrogen peroxide (H_2_O_2_) was used as a positive control. After incubation, cells were stained using 0.1% CV and observed under a microscope to validate the morphology of control and ASTA-treated HBECs (Priya and Pandian, 2020).

### 2.8 Molecular docking

The protein 3D structure of *S. mutans* and their virulence response regulators *GtfC* [Protein Data Bank (PDB) ID: 3AIC] and *VicR* (PDB ID: 5ZA3) were retrieved from the PDB database. The PubChem database was used to obtain the chemical structure of ASTA (PubChem CID: 119034). Virulence protein targets were prepared by AutoDock Tools Version 1.5.6, and the docking was performed using AutoDock (Trott and Olson, 2010). An analysis of the protein–ligand interaction and hydrogen bond formation was done by the Protein–Ligand Interface Profiler (PLIP) server.

### 2.9 *In vivo* infection studies with the *Danio rerio* (zebrafish)

#### 2.9.1 Ethical statement

All the experiments with *Danio rerio* were carried out according to the general guidelines of the Institutional Animal Ethics Committee, Alagappa University (Reg No: 2007/GO/ ReBi/S/18/CPCSEA dt 14.03.2018) and have authorized the *in vivo* experiments with the zebrafish model (Approval number: IAEC/AU/OCT2021/Fish-5).

#### 2.9.2 Zebrafish maintenance

Wild-type adult zebrafish (6 months old) were procured from Sridhar Aqua-farm, Chennai, India. The collected fish were acclimated in polysulfone tanks filled with fresh water at 28 °C for 24 h under a photoperiod of 14:10 h (light: dark cycle). All *in vivo* studies with zebrafish were analyzed in triplicate.

#### 2.9.3 Toxicity assessment

Toxicity of ASTA was determined by measuring the survival rate of fish in the presence of different concentrations of ASTA. For this assessment, six healthy fish were transferred into fresh aquaria containing ASTA at various concentrations, and the survival rate of experimental animals was calculated to be up to 96 h.

#### 2.9.4 Infection studies with *S. mutans*

In the experiment, healthy fish were infected with *S. mutans* by immersing the animals with bacterial suspension (1 × 10^6^ CFU/mL) in water for 12 h. Then, the *S. mutans-*infected fish were transferred into fresh aquaria containing ASTA and named as the treatment group, whereas fish that were transferred into buckets without ASTA were named as infected controls. On the other hand, non-infected animals were grouped as normal controls. Finally, the mortality in each group was assessed until 96 h, and the percentage of survival was estimated by counting the total number of dead animals in every group.

#### 2.9.5 CFU assay

CFU assay was performed to confirm the reduction of *S. mutans* intestinal colonization in the ASTA-treated animals. Following the infection experiment, from each group, three fish were taken and washed with PBS. The intestinal part of these fish was then finely crushed and suspended in the PBS solution. The crushed intestinal mixture was vortexed and centrifuged at 3000 rpm for 2 min. Finally, the suspension was serially diluted and plated on THYES agar plates to count the number of viable *S. mutans* cells present in the ASTA-treated and untreated fish sample.

#### 2.9.6 Histopathological examination

Histopathological analysis was performed to examine the systemic infections caused by *S. mutans* in zebrafish. After 3 days of infection, experimented fish were collected and fixed immediately with 10% phosphate-buffered formalin and decalcified with 0.5 M EDTA at 42 °C for 8 h. Furthermore, the samples were then dehydrated by using ethanol, cleared in xylene, and fixed in paraffin wax. The tissues were sectioned transversely at 5 μM using a rotary microtome and stained with eosin and hematoxylin. Finally, the samples were viewed under a light microscope (Nikon Eclipse Ti 100) at 400 × magnification.

### 2.10 Statistical analysis

For statistical analysis, SPSS statistics 17.0 was used (Chicago, IL, United States). To evaluate the significant differences among the control and treated samples, a one-way ANOVA with Duncan's *post-hoc* test was performed. All the experiments were carried out in at least three biological replicates with at least two technical replicates, and values are presented as mean ± standard deviation (SD). A *p-*value of < 0.05 was considered statistically significant.

## 3 Results

### 3.1 Determination of MIC and MBC

**Figure 1:**
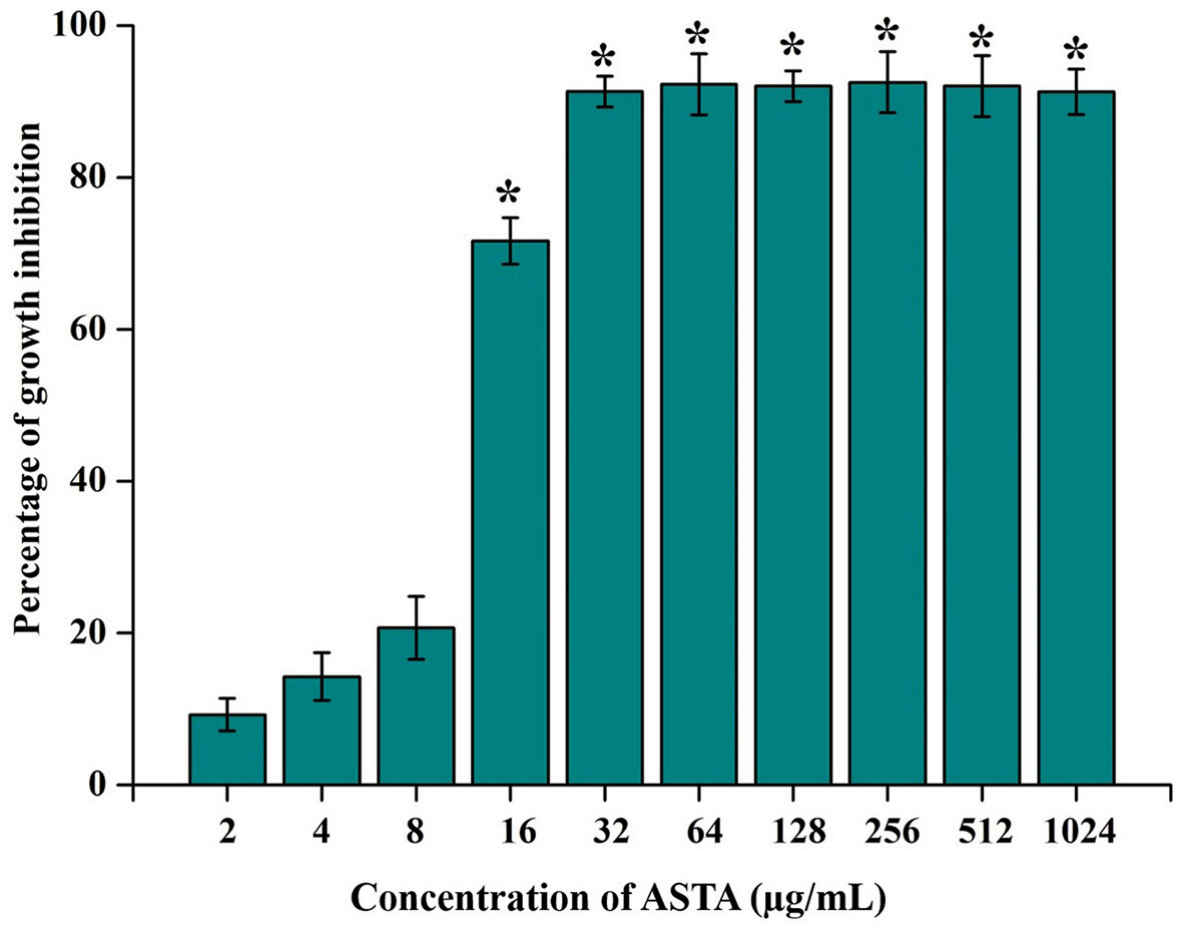
Effect of ASTA on growth of *S. mutans*. ASTA significantly inhibited the growth of *S. mutans* in a concentration-dependent manner. Data are expressed as mean ± SD (*n* = 3), and the asterisk indicates significant differences compared with the untreated control (*p* < 0.05).

**Figure 2:**
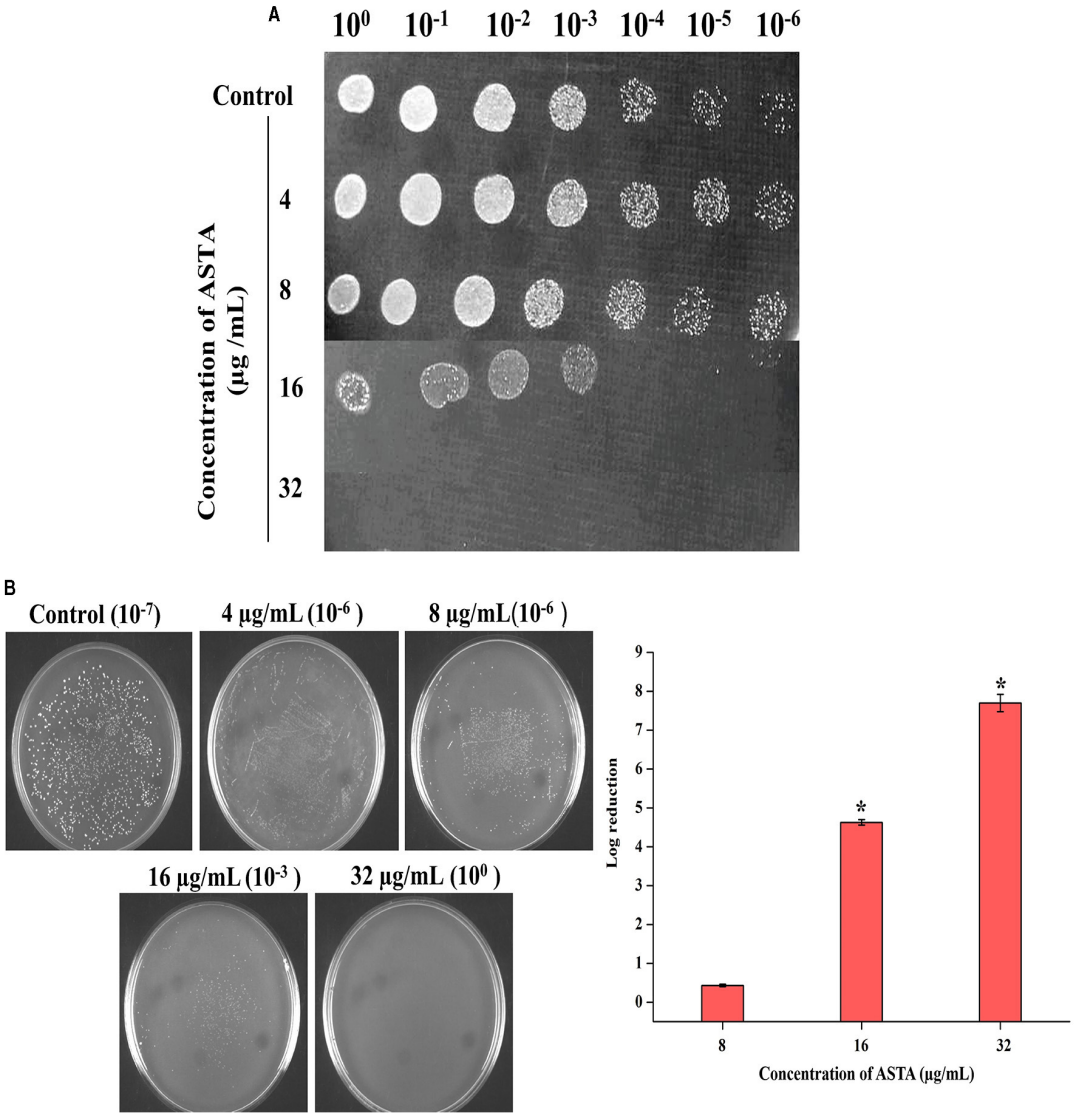
Bactericidal activity of ASTA on *S. mutans*. **(A)** Spot assay to validate the bactericidal activity of ASTA. **(B)** The MBC was determined through CFU assay. Data are expressed as mean ± SD (*n* = 3), and the asterisk indicates significant differences compared with the untreated control (*p* < 0.05).

The MIC of ASTA against *S. mutans* was analyzed in the range of 2–1064 μg/mL. At 32 μg/mL concentration of ASTA, complete growth inhibition was observed, and this concentration was determined as the MIC (Figure 1). No viable cells were detected at the 32 μg/mL concentration, while spot and spread plating was done without serial dilution (Figures 2A, B). Hence, the MBC of ASTA against *S. mutans* was 32 μg/mL.

### 3.2 Effects of ASTA on the *S. mutans* biofilm

#### 3.2.1 Quantification and microscopic visualization

**Figure 3:**
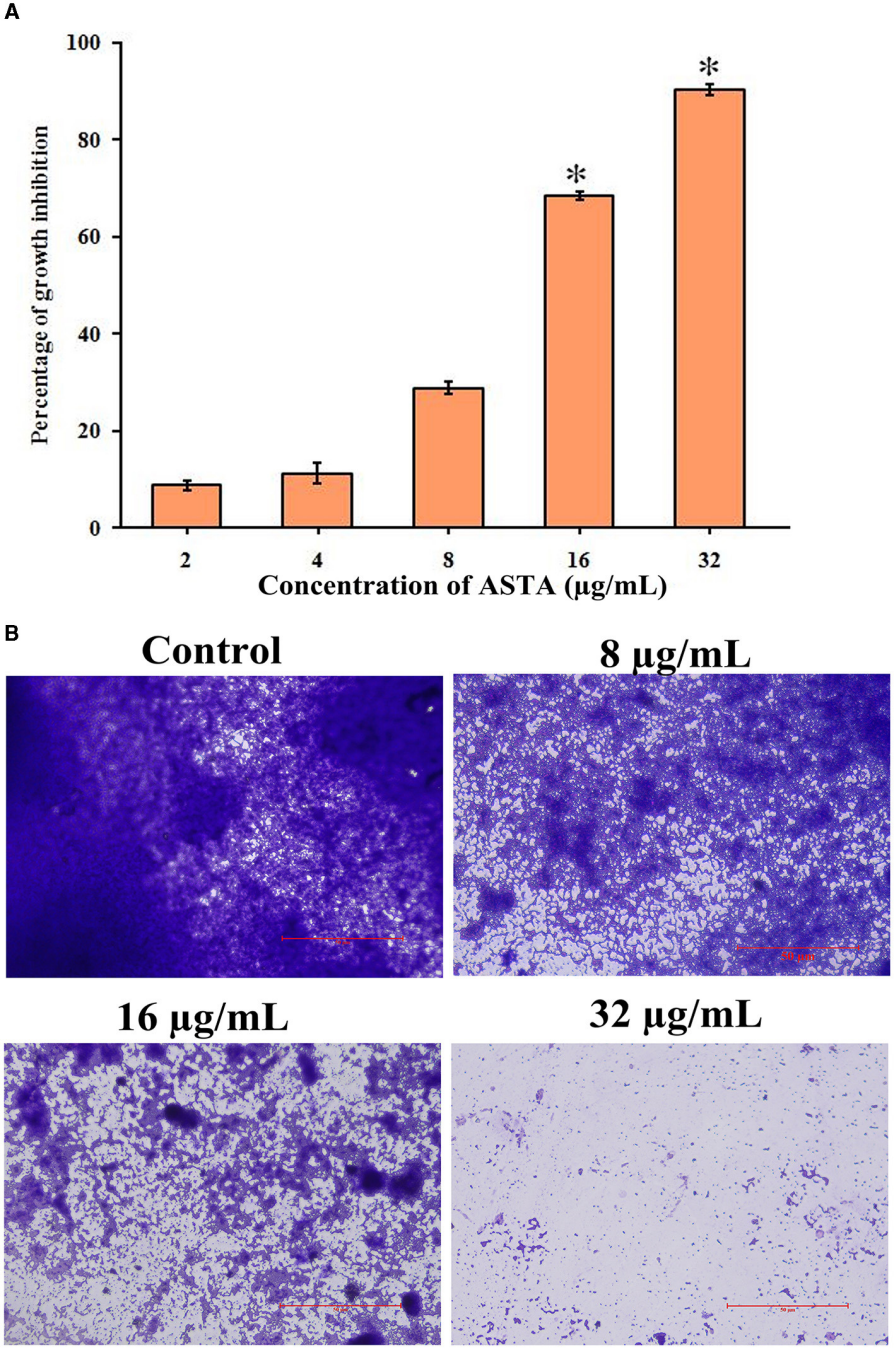
Effect of ASTA on biofilm formation. **(A)** Concentration-dependent reduction in formation of the surface-attached *S. mutans* biofilms was observed through the spectrophotometric method and **(B)** light microscopic visualization. The scale bar indicates 50 μm. Data are expressed as mean ± SD (*n* = 3), and the asterisk indicates significant difference compared with the untreated control (*p* < 0.05).

From the spectrophotometric and microscopic analyses, concentration-dependent reduction in the surface-attached biofilms was observed. From the microscopic analysis, it is evident that, compared to untreated controls, the number of multilayered biofilms was reduced in the ASTA-treated samples. At 16 μg/mL concentration, a noticeable decrease in *S. mutans* biofilms was observed under a light microscope (Figures 3A, B), demonstrating the antibiofilm potential of ASTA against *S. mutans*.

#### 3.2.2 Mature biofilm disruption

**Figure 4:**
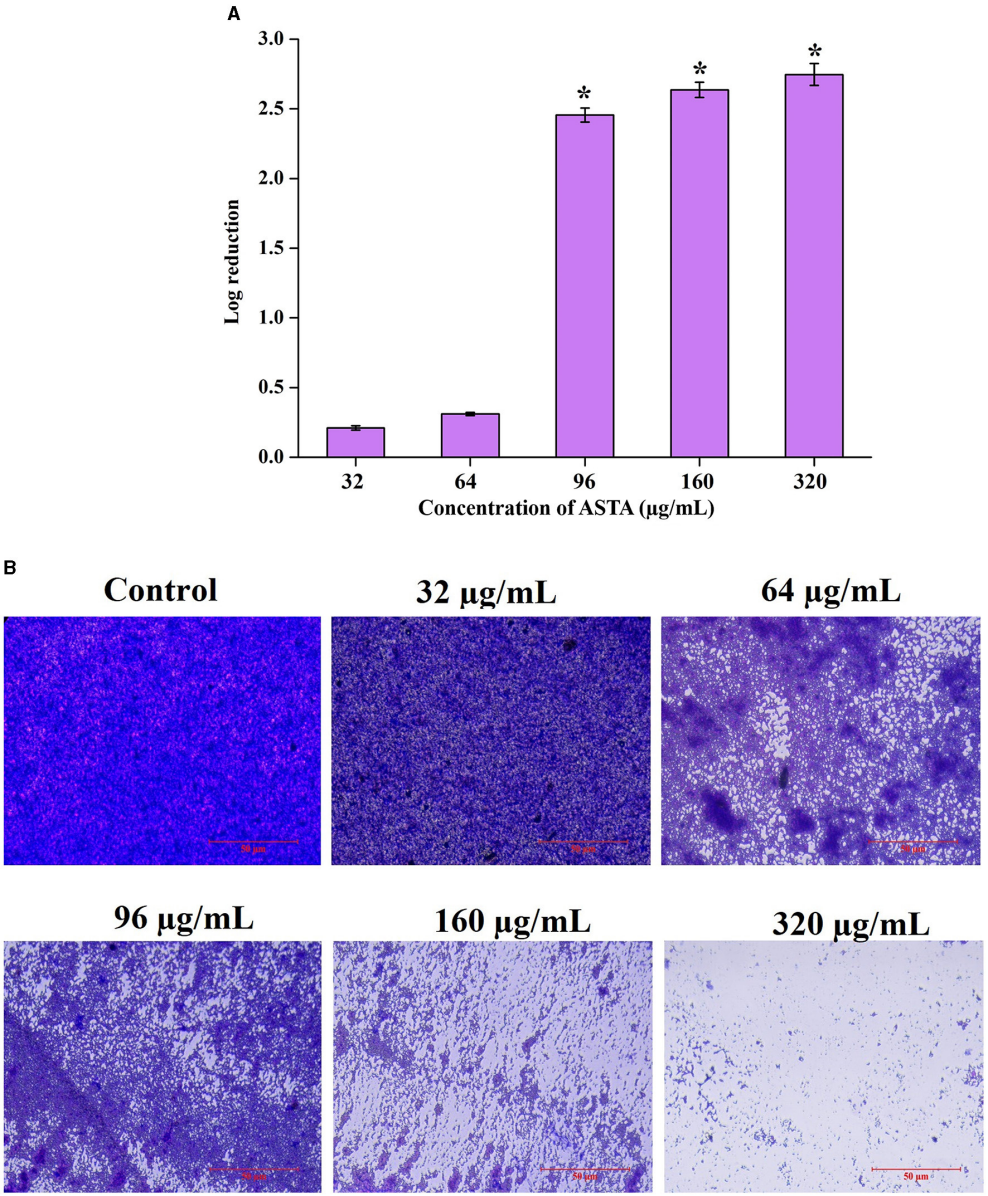
Mature biofilm disruptive efficacy of ASTA. *S. mutans* cells in the preformed biofilm and mature biofilm were observed to be affected by the treatment of ASTA. **(A)** Significant concentration-dependent reduction in the viable biofilm cells was observed through spectrophotometric and **(B)** microscopic visualization analyses. The scale bar indicates 50 μm. Data are expressed as mean ± SD. * indicates significant at a *p* ≤ 0.05.

The disruptive potential of ASTA on mature or preformed biofilms was determined by CFU and light microscopic analysis. Compared to untreated controls, ASTA-treated samples of mature biofilm cells showed significant reduction in viable cell numbers in the preformed biofilm. Moreover, microscopic analysis showed that ASTA effectively decreases the solid coating of biofilm cells when compared to the absence of ASTA (Figures 4A, B).

#### 3.2.3 Acidogenicity potential of ASTA against S. mutans

The most crucial virulence factor of *S. mutans* in the progression of dental carries in humans is the acidogenic potential. Acidogenic property is the potential of *S. mutans* to generate acid by-products through the fermentation of carbohydrates present in the food source. The glycolytic pH drop assay was performed to examine the effect of ASTA on the acidogenic property of the bacteria.

**Figure 5:**
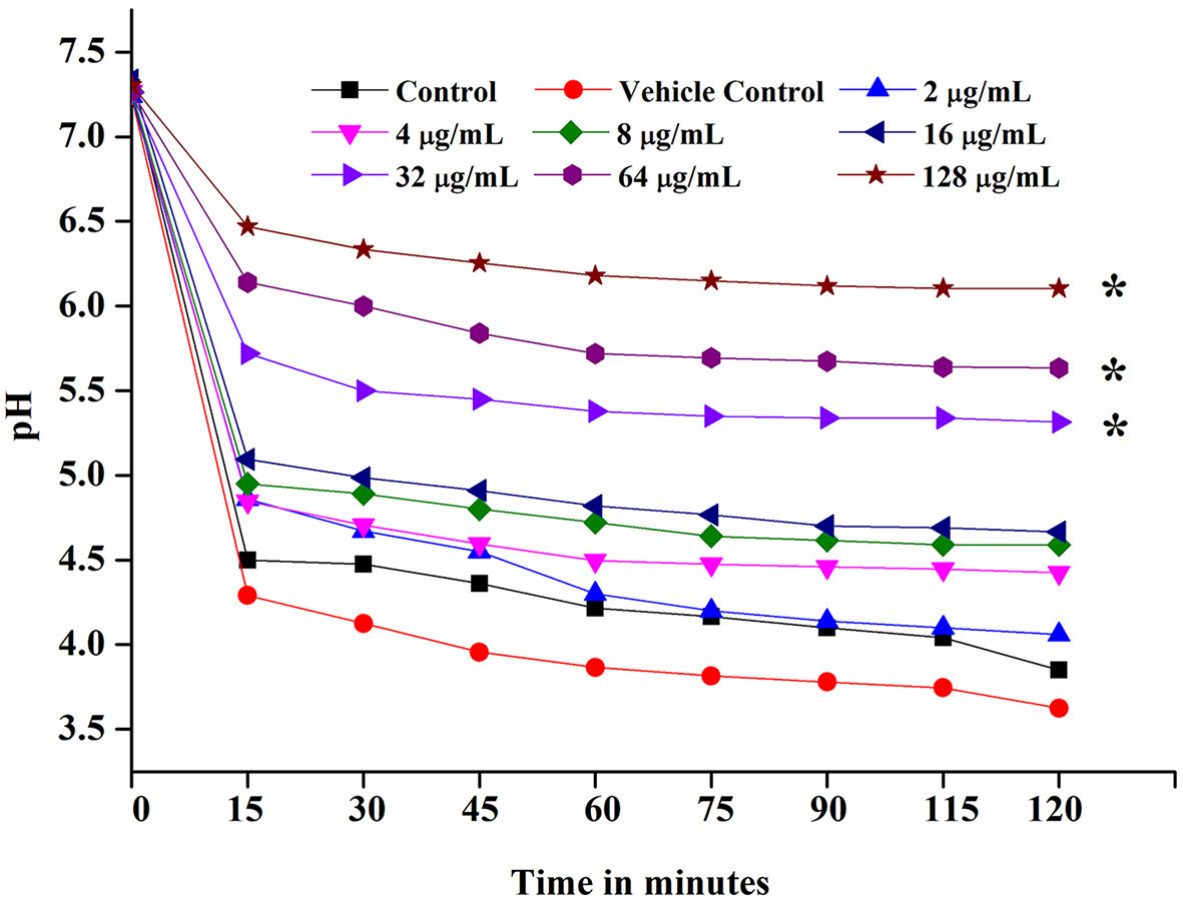
Glycolytic pH drop assay. ASTA significantly inhibited the glycolytic pH drop at MIC and MBC. Data are expressed as mean ± SD. * indicates significant at a *p* ≤ 0.05.

As a result, ASTA slows down the process of acidogenic properties of *S. mutans*, and pH was neutral at the 64 μg/mL concentration. This result can be evident from the graph in which the pH of the control reduced drastically from 7.2 to 4.5, whereas ASTA-treated samples slow down the process of acid conversion. The acidogenic potential of *S. mutans* was delayed by ASTA-treated samples when compared to the controls (Figure 5).

#### 3.2.4 Acidity measurement of the spent medium

**Figure 6:**
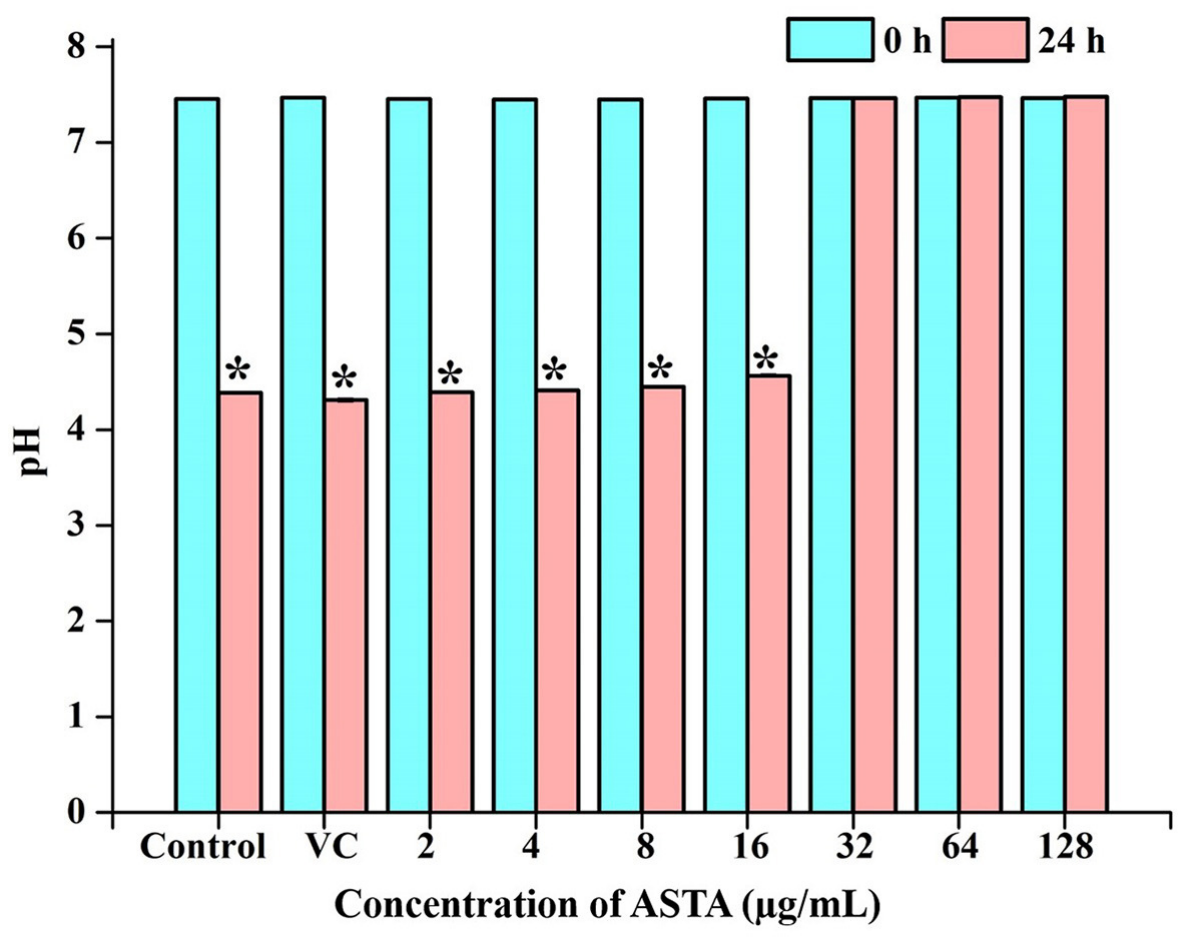
pH of the spent medium. Acidic nature of the broth was observed to be increasing with ASTA treatment. Data are expressed as mean ± SD. * indicates significant at a *p* ≤ 0.05.

Initial and pH after 24-h incubation of the *S. mutans* grown under the various concentrations of ASTA were observed. It is evident that medium pH was decreased in a concentration-dependent manner (Figure 6).

#### 3.2.5 Efficacy of ASTA on acid tolerance of *S. mutans*

**Figure 7:**
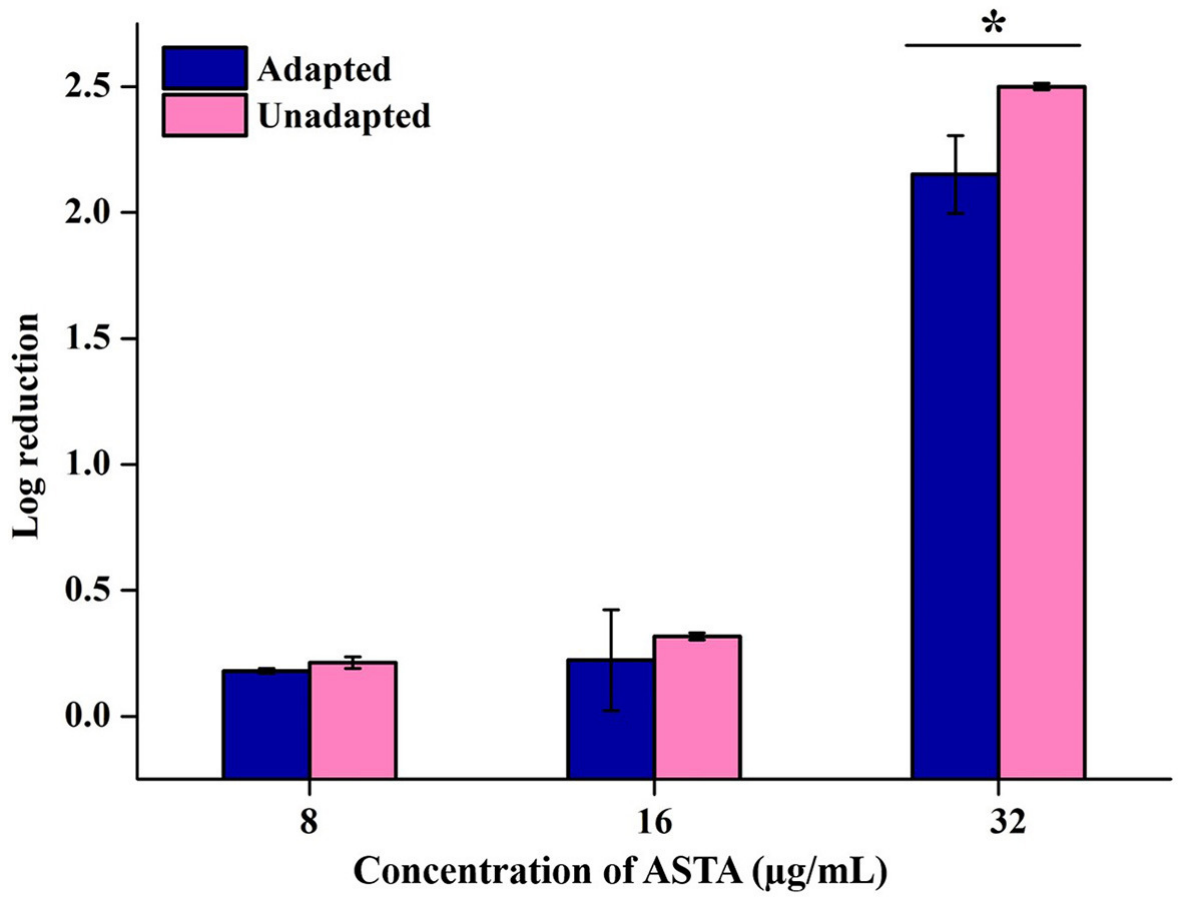
Effect of ASTA on the acid tolerance of *S. mutans*. ASTA effectively repressed the *S. mutans* cell survival rate at sub-MIC levels in the acidic condition compared to untreated controls. Data are expressed as mean ± SD. * indicates significant at a *p* ≤ 0.05.

The impact of ASTA on the acid tolerance potential of *S. mutans* was examined under the conditions of the adapted and unadapted group of cells. Unadapted cells were immediately cultured in adverse pH conditions, whereas adapted cells were initially subjected to mild pH conditions for the adaptation of *S. mutans* to acid conditions before being subjected to lethal pH. As a result, it is evident that, compared to unadapted cells, adapted cells showed increased survivability under lethal pH conditions. In addition, regardless of pre-adaptation to acidic conditions, while compared with controls, ASTA treatment reduced the number of viable cells under acidic conditions. This finding demonstrates that ASTA treatment may even be efficient on the *S. mutans* cells that are adapted to persist in an acidic environment (Figure 7).

#### 3.2.6 Potential of ASTA on eDNA content in the *S. mutans* biofilm

**Figure 8:**
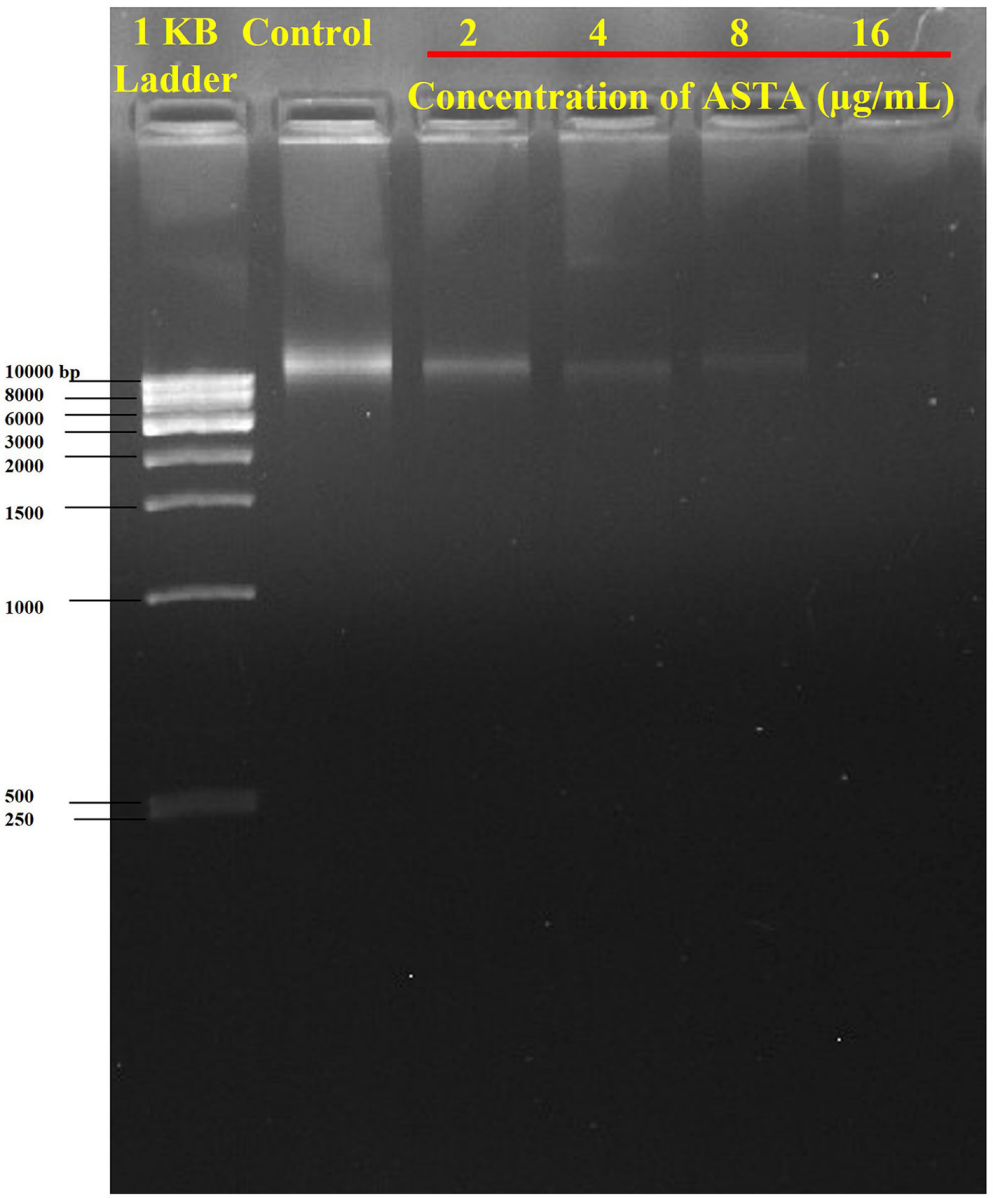
Inhibition of eDNA content in the ASTA-treated biofilm matrix.

The effect of ASTA on the eDNA content in *S. mutans* biofilms was qualitatively assessed by eDNA extraction accompanied by AGE. Notably, the amount of eDNA content was reduced in a concentration-dependent manner when compared with the controls. No visible band was detected at the concentration of 16 μg/mL. Thus, it is evident that ASTA effectively decreases the eDNA content in *S. mutans* biofilm cells (Figure 8).

### 3.3 Toxicity assessment on human buccal epithelial cells

**Figure 9:**
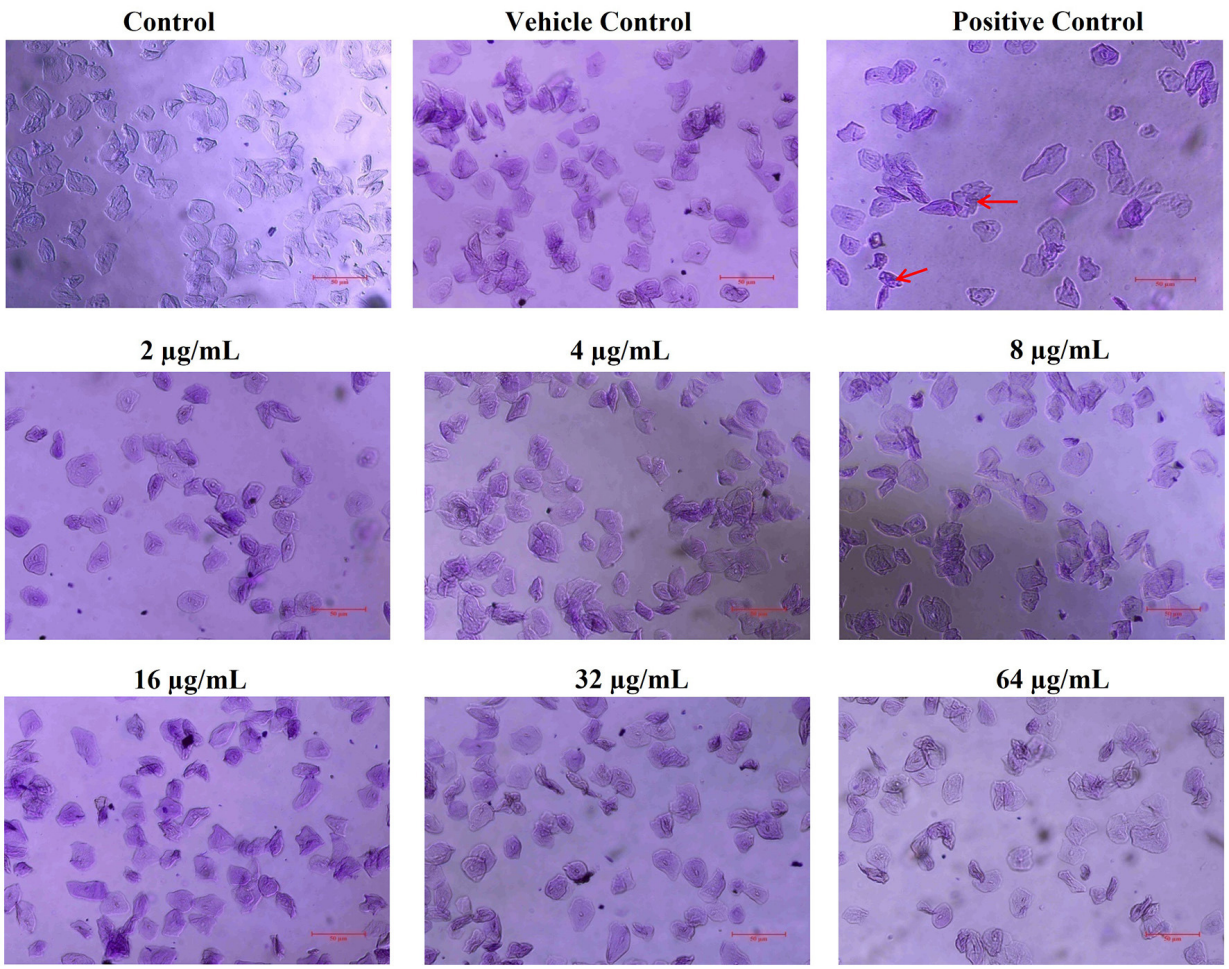
Nontoxic potential of ASTA to HBECs. ASTA-treated HBECs were found to be normal when compared to the control cells, and no morphological changes were observed. In contrast, H_2_O_2_-treated HBECs were observed to be damaged and devastated. The scale bar indicates 50 μm. Red arrows indicate damaged HBECs in positive control samples.

The safety prospects of using ASTA with dentifrice to treat dental plaque were assessed using HBECs. The microscopic analysis has shown that, similar to the untreated control, ASTA-treated HBECs were found to be healthy and normal. However, HBECs treated with positive control (H_2_O_2_) underwent morphological changes (Figure 9). This study revealed that ASTA is safe for oral application and is not toxic to HBECs.

### 3.4 Molecular docking of ASTA with GtfC and VicR

**Figure 10:**
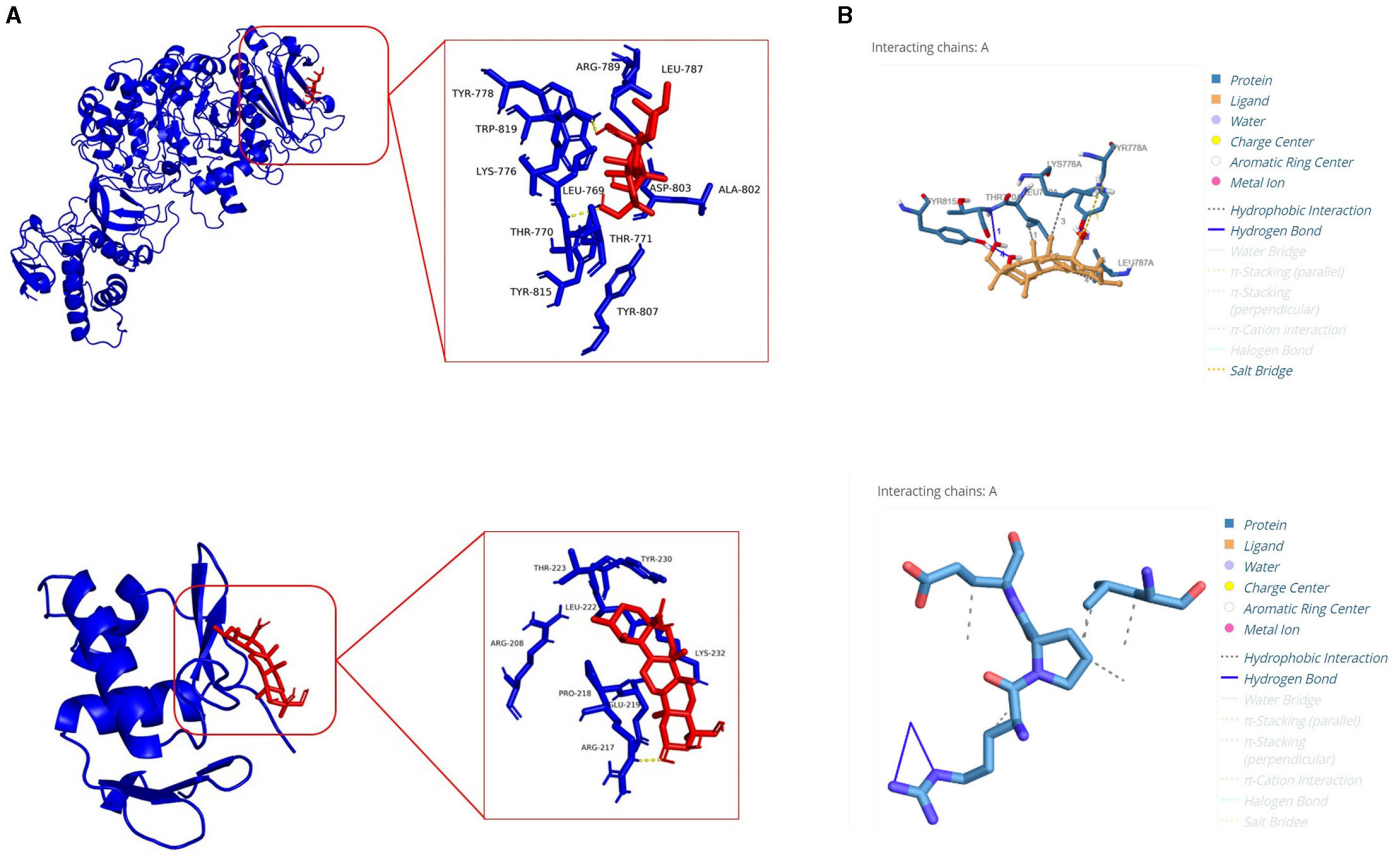
Molecular docking analysis. **(A)** Molecular interaction of ASTA with GtfC and VicR. **(B)** Protein–ligand interaction profiler analysis showing hydrogen bonds between amino acid residues of GtfC and VicR with ASTA.

The results of the molecular docking analysis revealed the ability of ASTA to interact with GtfC and VicR proteins of *S. mutans*. Significantly, ASTA interacts with the active sites of GtfC with the binding energy of −5.53 kcal/mol and hydrogen bonding with three amino acids (TYR778, TYR815, and THR770). The TYR778 amino acids form two hydrogen bonds with ASTA with the distance between them of 1.90 and 2.17 Å, whereas TYR815 and THR770 form one hydrogen bond with the ASTA of 2.24 and 2.58 Å, respectively. In case of VicR, ASTA actively interacts with ARG217, which has two hydrogen bonds of 2.25 and 3.45 Å, and the binding energy of ASTA with VicR was −4.75 kcal/mol (Table 1 and Figure 10). The molecular docking results suggested the possible interaction of ASTA with GtfC and VicR, and it strongly supports the obtained study results.

**Table 1:** Molecular docking analysis showed ASTA binding efficacy and hydrogen bond with GtfC and VicR of *S. mutans*.

**Receptor**	**Ligand**	**No of hydrogen bonds**	**Key residue**	**Binding energy (kcal/mol)**
GtfC	ASTA	4	THR A: 770 TYR A: 778 TYR A: 815	−5.53
VicR	ASTA	2	ARG A:217	−4.75

### 3.5 *In vivo* toxicity of ASTA in zebrafish

**Figure 11:**
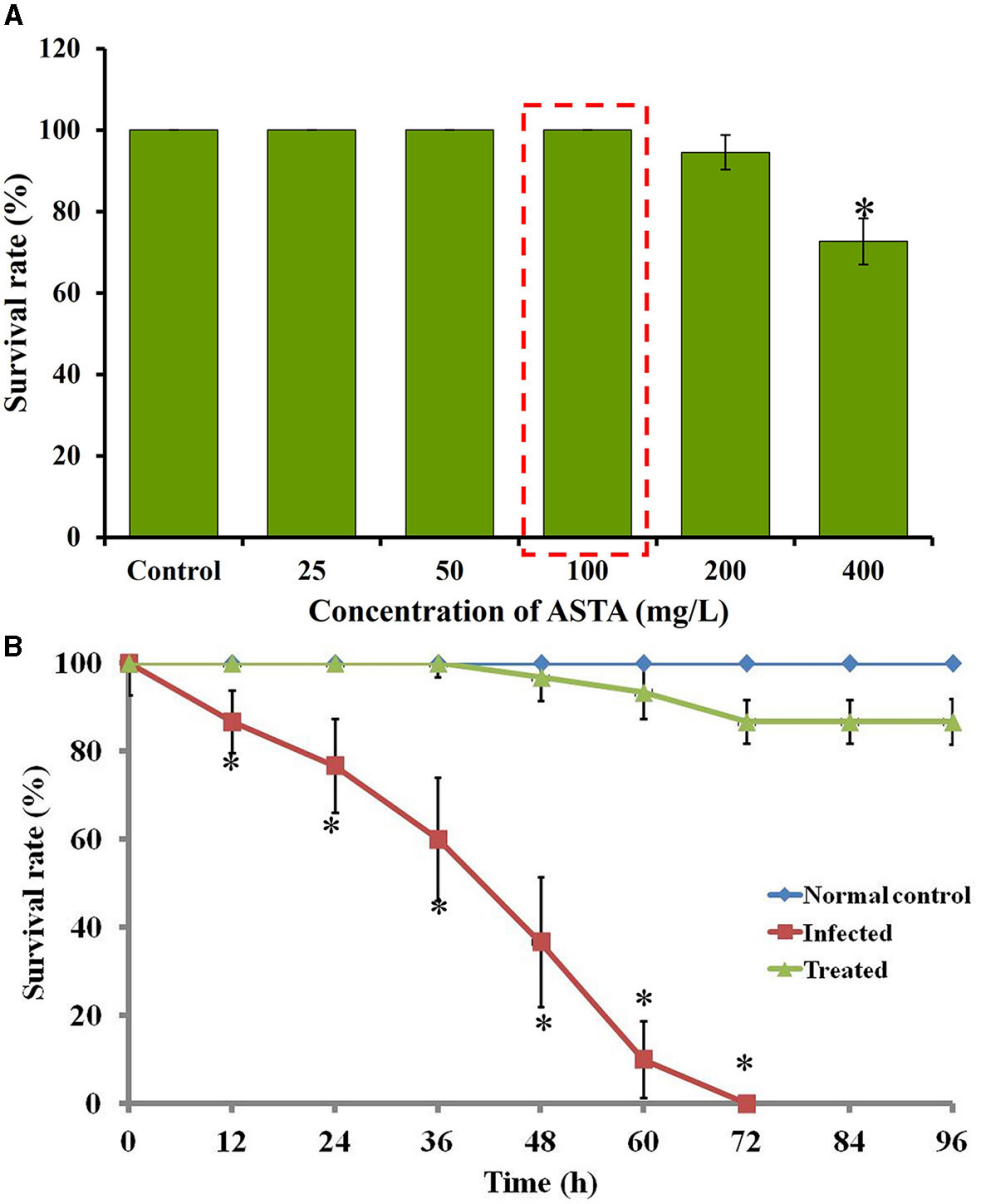
**(A)** The survival rate of ASTA-treated fish during toxicity assay. **(B)** Survival percentage of *S. mutans*-infected fish upon ASTA treatment at the 32 μg/mL concentration at up to 96 h. Data are expressed as mean ± SD. * indicates significant at a *p* ≤ 0.05.

The *in vivo* toxicity of ASTA was observed by evaluating the survival rates of fish cultured in the presence and absence of ASTA at different concentrations. The results revealed that the ASTA showed 100% survival at 32 μg/mL concentration (MIC). Hence, the same concentration was fixed for the *in vivo* anti-infection studies against *S. mutans* (Figure 11A).

### 3.6 Expansion in the lifespan of infected fish

The efficacy of ASTA against the *S. mutans* infection on zebrafish was determined by the survival assay with 12 h post-infected fish. In the *S. mutans* infection, the survival rate of infected fish was decreased to 63.33% after 48 h of post-infection, and complete mortality was observed within 72 h. However, the fish treated with ASTA (at 32 μg/mL) showed a survival rate of 100% even after 4 days of post-infection, indicating the anti-infective potential against *S. mutans* infection (Figure 11B).

### 3.7 Clinical symptoms of *S. mutans-*infected and treated zebrafish

**Figure 12:**
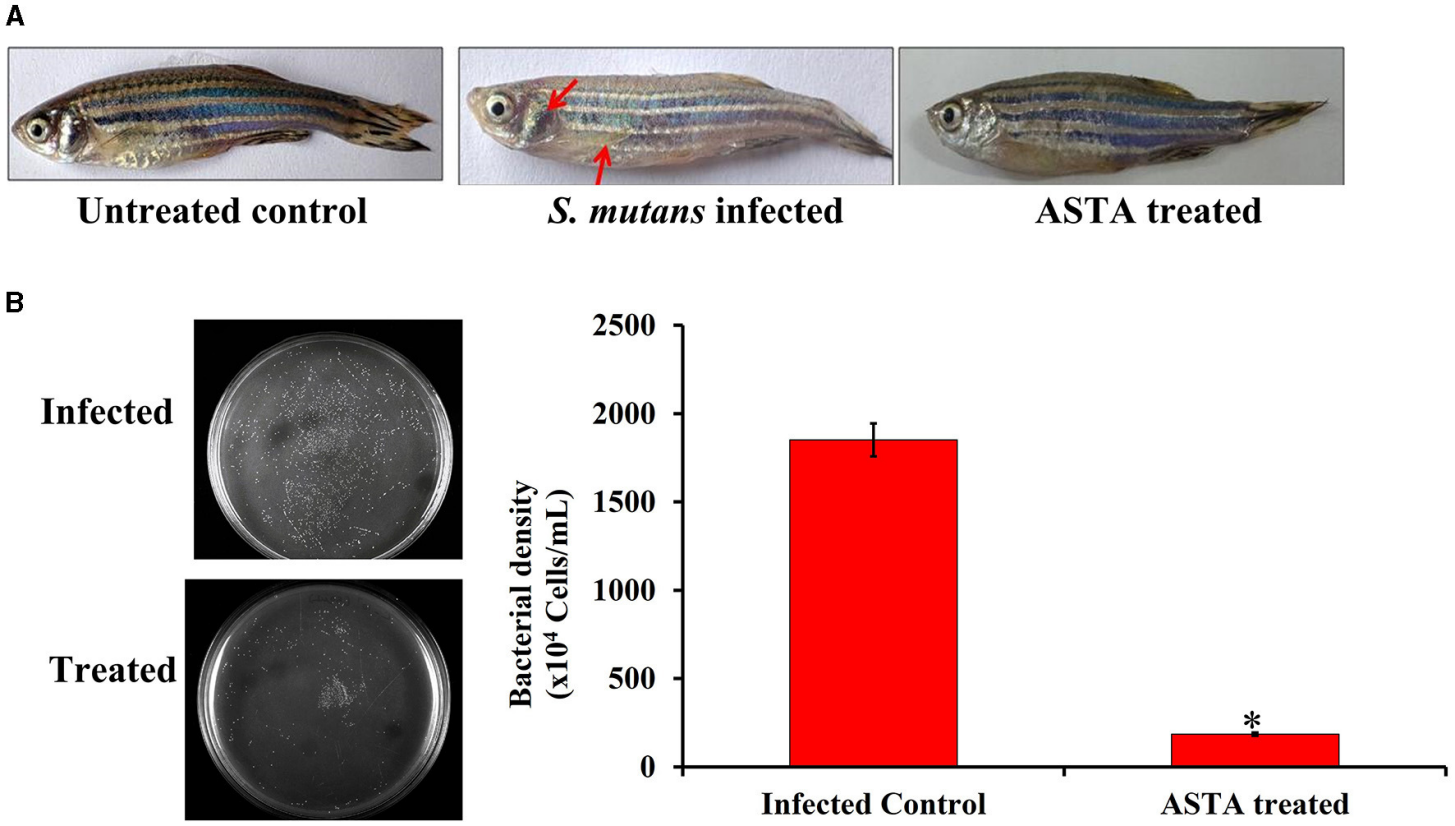
**(A)** Photographs showing the protective efficacy of ASTA treatment on clinically significant symptoms of *S. mutans*-infected fish **(B)** Furthermore, the result of CFU analysis showed the protective efficacy of ASTA treatment in reducing bacterial colonization of *S. mutans* in zebrafish. Data are expressed as mean ± SD. * indicates significant at a *p* ≤ 0.05.

During the *S. mutans* infection, various parts of the zebrafish such as the intestine, gills, and skin were severely infected. The occurrence of inflammation on the infected site revealed the severity of infection, as shown in Figure 12A. The redness and inflammation effects were not observed in ASTA-treated animals when compared to the infected controls. Additionally, treated animals exhibited more active swimming behavior than the infected controls. Moreover, the normal zebrafish skin showed the melanophore-rich dark stripes, whereas in infected control animals, abnormalities in the pigment coloration were observed with pale melanophores (Figure 12A). In case of ASTA-treated animals, the pigmentation of melanophores was recovered.

### 3.8 CFU analysis

*In vitro* CFU assay was performed to evaluate the effect of ASTA on *S. mutans* intestinal colonization in zebrafish using THYES agar. The results revealed that the ASTA treatment significantly reduced the intestinal *S. mutans* colonization compared with their control groups (Figure 12B).

### 3.9 Histopathology analysis

**Figure 13:**
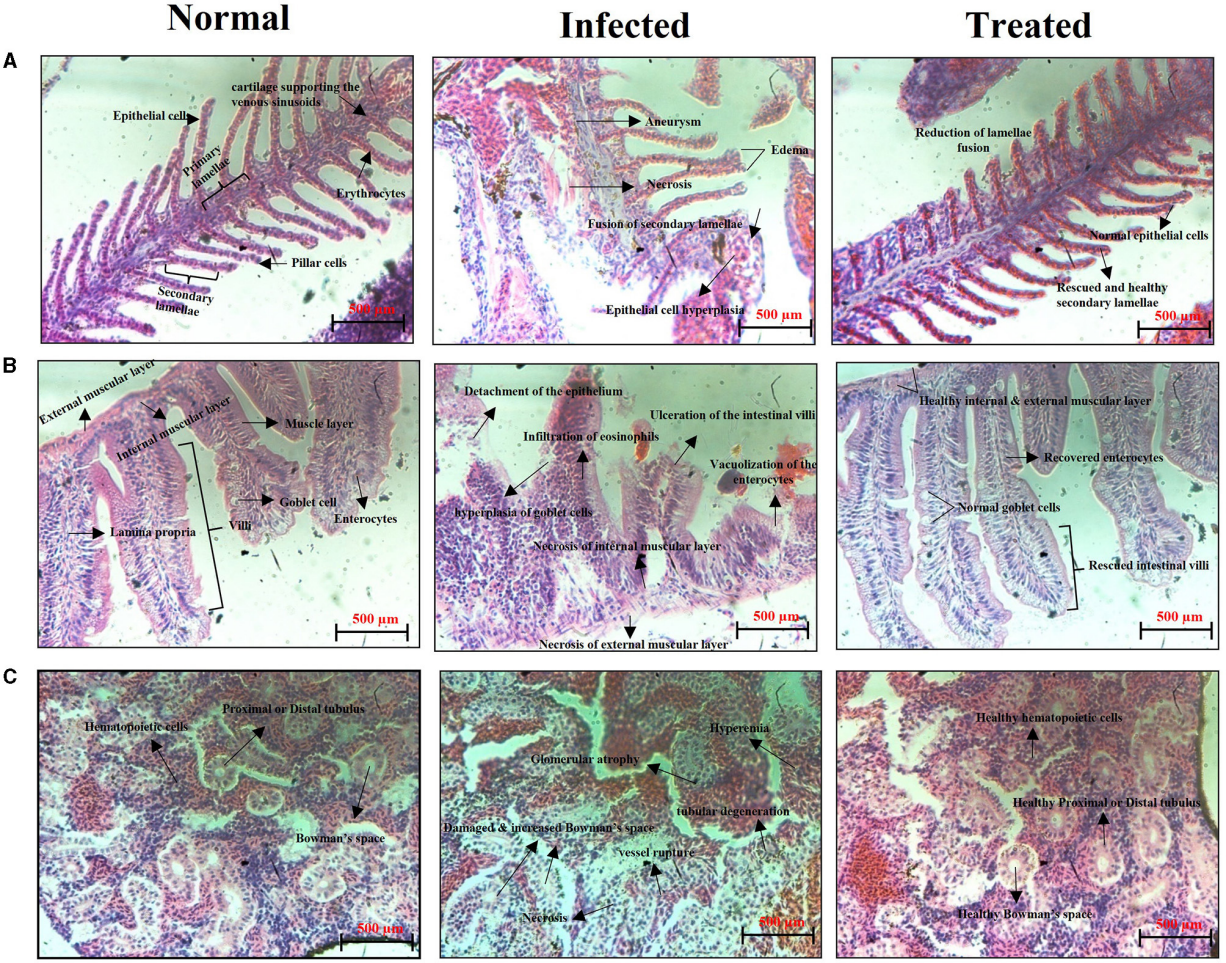
Histopathology observation of the gills **(A)**, intestine **(B)**, and kidney **(C)** sections of the normal uninfected control, *S. mutans-*infected control, and ASTA-treated zebrafish.

The *S. mutans-*infected and ASTA-treated zebrafish gills, intestine, and kidney samples were subjected to histopathological analysis. Figure 13 exhibits the histopathology of vital organs observed from the normal control, *S. mutans-*infected control, and ASTA-treated groups. The normal control group shows the healthy and regular structure of primary and secondary lamellae, epithelial cells, and pillar cells, whereas significant damages were observed in the *S. mutans-*infected control group. A histopathology analysis shows an increase in the size of epithelial cells (hyperplasia), a fusion of secondary lamellae, aneurysms, necrosis, gill tissue collapse, and edema (Figure 13A). This result reveals evidence of a respiratory infection caused by *S. mutans*. Interestingly, these pathognomonic damages of secondary lamellae fusion, cell degeneration in lamellae, displacement of the lamella epithelial cells, and hyperplasia were not observed in the ASTA-treated group, indicating the rescue potential of ASTA against the *S. mutans* infection. The histopathology of the intestine of normal controls exhibited healthy and normal intestinal villi, intestinal goblet cells, lamina propria, enterocytes, and the external and internal mucosal layer without any noticeable pathological changes. However, the infected animal group showed hyperplasia of goblet cells, intestinal and external mucosal layer degeneration, detachment of the epithelium, epithelium enlargement, severe eosinophil infiltration in the tissue, vacuolization of the enterocytes, and ulceration of the intestinal villi. Notably, zebrafish treated with ASTA on post-infection revealed some organelle damages, with significant rescue after treatment (Figure 13B). The kidney of the normal control group shows healthy hematopoietic tissue and renal tubules, whereas the kidney of the infected animal had lost its normal structure and showed an enlarged intestinal space between the rental tubules and glomeruli, tubular degeneration, vessel rupture, necrosis of hematopoietic tissue, and glomerulus atrophy. Interestingly, these damages were significantly recovered in the treated animals after ASTA treatment (Figure 13C).

## 4 Discussion

The efficacy of ASTA derived from plants in comparison to its chemically synthesized counterparts in the treatment of diseases may exhibit variability based on various factors such as purity, bioavailability, formulation, and potential synergies with other compounds found in the plant extract. Natural sources contain supplementary phytocompounds, antioxidants, and bioactive substances that can improve the pharmacological properties of ASTA through synergistic interplays (Lv et al., 2018). Plant-derived ASTA may demonstrate enhanced pharmacological efficacy due to its natural composition and structure (Oboh et al., 2021). It contains various biologically active components, including triterpenoids, flavonoids, and polyphenols, that collectively enhance its therapeutic effectiveness for the treatment of various diseases. In traditional medical systems such as Ayurveda and Traditional Chinese Medicine, the utilization of plant-based ASTA has spanned centuries for the purpose of addressing a treatment of various diseases. The traditional knowledge and practices may provide significant enlightenment regarding the therapeutic properties and ideal application of plant-derived asiatic acid for particular health conditions. The current investigation intended to evaluate the antibacterial and anti-infective activity of Bacopa-derived ASTA against *S. mutans. S. mutans* is one of the major infectious substances that cause human dental caries, and it forms the biofilms on the abovementioned human solid tissues of the oral cavity (Zeng et al., 2019). There is an urgent need for the treatment and prevention of tooth decay with harmless and effective strategies. Hence, in the last few years, researchers focused on naturally derived plant products for the treatment of oral diseases (Palombo, 2011). For this study, ASTA was chosen for evaluating its antibacterial potential against *S. mutans*. ASTA showed MIC and MBC at 32 μg/mL concentration. MBC results strongly confirmed that, at the concentration of 32 μg/mL, complete inhibition of *S. mutans* was observed (Figures 1, 2). However, ASTA at sub-MIC levels effectively inhibits the proliferation of *S. mutans*. Hence, the results strongly recommend that ASTA is a promising antibacterial agent to treat the *S. mutans* infection.

*S. mutans* is a major component of human oral plaque biofilms that develop on the tooth surface (Loesche, 1986). Nearly 65% of human infectious diseases are examined to be a connection of microbial biofilms (Banas, 2004). The formation of dental plaque on the human tooth surfaces is a typical example of *S. mutans* biofilms (Samaranayake, 2012). The efficacy of ASTA at sub-MIC values on the virulence attributes of dental plaque by *S. mutans* biofilm was analyzed. Biofilm quantification results showed that ASTA significantly inhibited the biofilm formation at the level of 77% at a concentration of 16 μg/mL (Figure 3A). From this result, it can be confirmed that, even at the lowest concentration of ASTA, effective inhibition in the biofilm formation is observed, and it ultimately can reduce the formation of dental plaque. Furthermore, light microscopic images of *S. mutans* biofilms clearly revealed that, compared to untreated control slides, the depth of biofilms architecture was effectively decreased in the ASTA-treated slides (Figure 3B). The results confirmed that, unlike synthetic drug/antibiotics, inhibition of biofilms caused through ASTA is unrelated to planktonic cell growth inhibition.

Acidogenicity and acid tolerance are other important virulence attributes of *S. mutans* for tooth surface destruction, tooth enamel, and dental caries development (Banas, 2004; Guo et al., 2015). A glycolytic pH drop assay was executed to explore the impact of ASTA on acid production by *S. mutans*. Glycolysis is a major pathway for the production of acid. *S. mutans* can use a wide range of dietary sucrose and to produce acids. From the sucrose *S. mutans* synthesis, insoluble exopolysaccharide (EPS) and the EPS can distribute within the oral biofilms for adhesion and colonization of bacteria to hard tooth surfaces (Kim et al., 2015; André et al., 2017). The critical pH of tooth enamel ranged between 5.0 and 5.5, and if below the critical pH, destruction of tooth hard tissues and development of dental caries take place (Pandit et al., 2013). Our findings revealed that the initial value of pH slowly decreased, while increase in the ASTA concentration and the final pH rate were greater than the critical pH (Figure 5). Hence, it is confirmed that this compound significantly inhibits the acid production in a concentration-dependent manner, and this compound undoubtedly can prevent tooth demineralization, likely due to its ability to inhibit the glycolytic enzyme for acid production. *S. mutans* has the ability to survive under low acidic pH conditions to establish dental caries and tooth erosion progression (Forssten et al., 2010). Acidurity results showed that ASTA has the potential to decrease the survival chances of *S. mutans* cells under acidic pH conditions (Figure 7). Hence, it is evident that ASTA has a prominent impact on the acidurity and acidogenicity properties of *S. mutans*.

Furthermore, *S. mutans* is known to form biofilms that depend on eDNA when the pH is low. eDNA is a scaffolding extracellular matrix present in *S. mutans* biofilms. eDNA plays a crucial role in *S. mutans* biofilms such as the adhesion, stability, and development of *S. mutans* biofilms to the surface of tooth, facilitating formation of acidic environments and assisting in cell–cell communication (Klein et al., 2010). Research in *S. mutans* demonstrated that eDNA can be released in response to the competence-stimulating peptides (CSP) and SigX-inducing peptides (XIPs), which can induce autolysis and significantly influence the biofilm formation (Perry et al., 2009; Wenderska et al., 2012). It is striking that, compared to untreated control, ASTA-treated samples showed concentration-dependent reduction of the eDNA content in *S. mutans* biofilms at sub-MIC levels (Figure 8). From this result, it can be concluded that ASTA has the ability to repress the eDNA content in bacterial biofilms, and it ultimately prevents the adhesion and formation of biofilms to the tooth surface. Moreover, ASTA effectively acted on the preformed *S. mutans* mature biofilms. However, ASTA is a potential antibacterial drug for the treatment of *S. mutans* infection; it is necessary to test the toxicity of ASTA against HBECs. Toxicity testing is essential to develop novel drugs that can help protect human health from the negative consequences of environmental agent exposures (Krewski et al., 2010). ASTA was determined to be safe for HBECs, which confirmed its therapeutic potential for the treatment of *S. mutans* infection (Figure 9). In addition, molecular docking results of ASTA exhibited the crucial interaction with the high binding affinities of virulence factors VicR and GtfC. It strongly suggests that the ASTA potentially dysregulates the *S. mutans* virulence genes (Figure 10). Altogether, the *in vitro* findings demonstrated that ASTA is a suitable therapeutic drug against human oral pathogen *S. mutans*.

In addition, it is an important factor for a compound to have non-toxic nature for clinical applications. Various *in vitro* investigations have documented the inhibitory effects of ASTA on the growth, biofilm formation, and production of virulence factors by *S. mutans*, indicating that ASTA acted as a natural agent in the prevention of dental caries (Sycz et al., 2022; Nurhapsari et al., 2023). Nevertheless, further research such as *in vivo* examinations and clinical experiments are imperative to confirm these findings and evaluate the viability of interventions utilizing ASTA for enhancing oral health. However, *in vivo* studies employing animal models such as zebrafish play a crucial role in the identification of possible treatments for the *S. mutans* infection due to their ability to offer a comprehensive understanding of toxicity, drug pharmacokinetics, host–pathogen interactions, and applicability to human biology. These investigations serve to bridge the gap between preclinical studies and clinical application, ultimately culminating in the advancement of safe and efficacious therapies for infectious diseases. To date, there has been a lack of *in vivo* studies exploring the impact of ASTA on the *S. mutans* infection. Therefore, the current study aimed to study the *in vivo* therapeutic potential of ASTA against the *S. mutans* infection using zebrafish as a model organism. Zebrafish was considered as an ideal vertebrate model to examine various human diseases and therapeutic approaches due to its high anatomical similarity to humans (Gomes and Mostowy, 2020). Zebrafish and humans have 70% gene similarity, and 84% of which is associated with human disease-related genes. Unlike mice models, zebrafish are small in size, easy to handle, have low maintenance costs, and short generation time. Interestingly, eight drugs examined using zebrafish have advanced in clinical applications (Cassar et al., 2019). As a result, it has emerged as an eminent model for studying various infectious diseases and for the development of novel drugs. In the present study, the toxicity results showed that there is no reduction in the survival rate of ASTA (32 μg/mL)-treated animals, which showed 100% survival like normal controls, indicating the non-toxic nature of ASTA (Figure 11). On the other hand, ASTA treatment rescued the *S. mutans-*infected animals, which is executed by inhibiting the internal colonization of *S. mutans* and thereby enhanced the survival of infected animals (Figure 12).

Generally, fish pathogens infect their host through the skin, gills, gastrointestinal tract, and kidney, and these organs are particularly susceptible to attachment and colonization by bacterial species, which can cause severe infection (Li et al., 2019). Zebrafish organs have been exploited in several studies; among them the kidney, intestine, and gills are the major and vital organs associated with bacterial infections. Gills play a crucial role in the respiration process, and it is the primary site of microbial colonization (Griffitt et al., 2009). The highly toxic substances in the water are readily absorbed by gills. In light of this fact, gills are a crucial organ to conduct histopathology analysis when it absorbs the chemical substances by immersion (Borges et al., 2019). *S. mutans* colonization caused severe damages in zebrafish gills on the third day post infection. The infected group showed morphological abnormalities, lamellar fusion, progressive distension of the pillar cells leading to aneurysms and collapsed gill filaments, epithelial cell hyperplasia, and fusion of some secondary lamellae that caused decreased breathing ability, respiratory dysfunction, and suffocation (Campagna et al., 2008). The alteration in the gills function can increase the size of epithelial cells (hyperplasia); as a result, secondary lamellae fusion can occur. Due to this event, the passage of blood and water was blocked to reduce the work overload on lamellae cells. This results in an oxygenation deficit in the animal, which may cause death (Macirella and Brunelli, 2017; Manjunatha et al., 2022). However, in the primary and secondary lamellae structures, epithelial cells were found to be normal like normal controls, indicating the protective effect of ASTA against the *S. mutans* infection. Similar to mammals, zebrafish have a mucous layer of columnar epithelial tissue developed by enterocytes around the villi in the gastrointestinal tract (Wang et al., 2010). Even though they lack a stomach, the intestinal bulb, which is the anterior section of the intestine, serves as a substitute for the stomach. The role of this organ includes nutrient absorption and digestion. Hence, histopathology of the zebrafish intestine can become a promising tool to evaluate the safety aspects of orally administered phytocompounds (Carvalho et al., 2018). The infected group intestine histopathology showed enlarged goblet cells, collapsed lamina propria, and infiltration of eosinophils (Figure 13A). Inflammation causes infiltration of eosinophils into the tissue, which contributes to increased hyperemia (Carvalho et al., 2018). One more feature noticed in the enterocytes upon *S. mutans* infection is vacuolization, often accompanied by edema. The latter is often accompanied by degeneration of the internal and external muscle layers and villi (Figure 13B). These tissue damages hinder the nutrient absorption process, which often precedes necrosis. Histopathological images of the ASTA-treated group intestine showed rescued potential against the *S. mutans* infection, which strongly suggested that ASTA can be considered safe for oral application, and it has the ability to rescue the *S. mutans* infection. The kidney is one of the major organs affected by pathogens, and it has similarities with mammals (Meron et al., 2020). The most common tissue alterations in the kidney are found in the tubules and can also have an adverse effect on the function of kidney glomeruli. This effect may lead to cause glomerulus atrophy or degeneration, as can be seen by the increase in Bowman's space, and these tissue damages can result in necrosis of the kidney (Takashima and Hibiya, 1995), which are also noticed in *S. mutans-*infected animals' histopathological images. Conversely, the successful recovery of these damages in the kidney of the ASTA-treated group was observed (Figure 13C). Overall, the results of the present study found that ASTA could be a promising antibacterial agent to treat *S. mutans* infections and has the potential to be used as a therapeutic agent to treat dental caries caused by *S. mutans*.

## 5 Conclusion

In conclusion, the present study confirmed that ASTA significantly inhibited the growth of *S. mutans* in a concentration-dependent manner. Furthermore, production of the virulence factors, including acid production, acid tolerance, and eDNA synthesis, was significantly inhibited after ASTA treatment at the MBC concentration. As a result, ASTA as a plant-based natural product may help influence antibacterial potential against the *S. mutans* biofilm. Additionally, the *in vivo* results unveiled the anti-infective efficacy of ASTA by protecting animals against the pathogenicity of *S. mutans*. Therefore, our study offers an effective alternative strategy for the prevention and treatment of dental plaque (or) oral biofilm and dental caries.

Furthermore, this study hypothesizes that integrating this plant extract with the nanoparticle technology holds significant potential for exploring novel avenues in preventing and treating dental caries. Encapsulation of plant-derived phytocompounds within nanoparticles will increase their efficacy and facilitate precise delivery (which yet remains to be studied). This strategy optimizes the therapeutic properties of plant extracts while reducing the potential side effects. Incorporating nanoparticles synthesized from plant extracts into various dental products including toothpaste, dental coatings, mouthwashes, and restorative materials is an innovative approach for the prevention of caries, the promotion of remineralization, and the maintenance of oral hygiene. In addition, advanced gene intervention techniques like CRISPR-Cas9 can be employed for the alteration of the oral microbiome, ultimately leading to a decrease in the occurrence of cariogenic bacteria. By focusing on genes crucial for bacterial virulence or biofilm formation, these methods have the potential to prevent the development and progression of dental caries. Moreover, the utilization of gene editing techniques may also be used to improve the host defense mechanism against oral pathogens, thereby fostering better oral hygiene. Overall, the integration of plant extracts with nanoparticle technology and emerging gene intervention techniques signifies a frontier of innovation in oral healthcare. Further research and collaboration among various fields will play a vital role in transforming these futuristic perspectives into practical advancements that improve patient outcomes and modernize dental care.

## Data availability statement

The original contributions presented in the study are included in the article/supplementary material, further inquiries can be directed to the corresponding author.

## Ethics statement

The studies involving humans were approved by the Institutional Ethical Committee, Alagappa University, Karaikudi (IEC ref no.: IEC/AU/2018/5). The studies were conducted in accordance with the local legislation and institutional requirements. The participants provided their written informed consent to participate in this study. The animal study was approved by Institutional Animal Ethics Committee, Alagappa University (Reg. no.: 2007/GO/ ReBi/S/18/CPCSEA dt 14.03.2018). The study was conducted in accordance with the local legislation and institutional requirements.

## Author contributions

RJ: Conceptualization, Data curation, Formal analysis, Investigation, Methodology, Validation, Visualization, Writing – original draft. PM: Conceptualization, Data curation, Formal analysis, Investigation, Methodology, Validation, Writing – review & editing. AP: Formal analysis, Investigation, Methodology, Validation, Writing – review & editing. RA: Investigation, Methodology, Validation, Writing – review & editing. NRSS: Data curation, Methodology, Formal analysis, Validation, Investigation, Visualization, Software, Writing – review & editing. SN: Formal analysis, Methodology, Validation, Writing – review & editing. HS: Formal analysis, Writing – review & editing. SKP: Formal analysis, Investigation, Methodology, Writing – review & editing. AVR: Formal analysis, Investigation, Methodology, Writing – review & editing. MR: Conceptualization, Data curation, Formal analysis, Methodology, Project administration, Supervision, Validation, Writing – review & editing.
